# Radiomics for Predicting the Development of Brain Edema from Normal-Appearing Early Brain-CT After Cardiac Arrest and Return of Spontaneous Circulation

**DOI:** 10.3390/diagnostics15020119

**Published:** 2025-01-07

**Authors:** Michael Scheschenja, Eva-Marie Müller-Stüler, Simon Viniol, Joel Wessendorf, Moritz B. Bastian, Jarmila Jedelská, Alexander M. König, Andreas H. Mahnken

**Affiliations:** Clinic of Diagnostic and Interventional Radiology, Marburg University Hospital, Philipps-University Marburg, Baldingerstrasse, 35043 Marburg, Germany

**Keywords:** cardiac arrest, return of spontaneous circulation, hypoxic-ischemic brain injury, radiomics, machine learning

## Abstract

**Background:** Hypoxic-ischemic brain injury (HIBI) is a feared complication post-cardiac arrest (CA). The timing of brain imaging remains a topic of ongoing debate. Early computed tomography (CT) scans can reveal acute intracranial pathologies but may have limited predictive value due to delayed manifestation of HIBI-related changes. Radiomics analyses present a promising approach to identifying subtle imaging markers, potentially aiding early HIBI detection. **Methods:** This study retrospectively assessed post-CA patients between 2016 and 2023 who received immediate brain CTs. Patients without acute intracranial pathology on initial scans and who underwent follow-up brain CTs within 14 days post-return of spontaneous circulation (ROSC) were included. Image segmentation involved manual basalganglia segmentation and automated whole-brain segmentation. Radiomics features were calculated using Pyradiomics (v3.0.1) in 3DSlicer (v5.2.2). Feature selection involved reproducibility analysis via ICC and LASSO regression, retaining five features per segmentation method. A logistic regression model for each segmentation method underwent 5-fold cross-validation. Results were summarized with ROC analyses and average sensitivity and specificity. **Results:** A total of 83 patients (average age: 65 ± 13.3 years, 19 women) with CA and ROSC were included. Follow-up CT scans after 5.2 ± 2.9 days revealed brain edema in 47 patients. The model using manual segmentation achieved an average AUC of 0.76, sensitivity of 0.59, and specificity of 0.78. The automated segmentation model showed an average AUC of 0.66, sensitivity of 0.49, and specificity of 0.68. **Conclusions:** Radiomics, particularly focused on the basalganglia area in normal-appearing brain CTs after CA and ROSC, may enhance predictive insights for HIBI and the development of brain edema.

## 1. Introduction

Cardiac arrest (CA) represents a critical medical emergency, with profound implications for multiple organ systems. Among the myriad complications associated with CA, the risk of damage to vital organs, particularly the brain, stands paramount. Even if survived, a relevant and feared complication is hypoxic-ischemic brain injury (HIBI), which may lead to severe neurological disabilities or even death [1,2,3,4].

The development of HIBI is a complex phenomenon linked not only to various contributing factors but also manifesting and progressing over a timeframe spanning from hours to several days [1,4,5]. Early identification of HIBI is important for clinical decision-making, including timely initiation of neuroprotective interventions, intensified monitoring, or withdrawal of life-sustaining treatments. Brain imaging, therefore, plays a vital role in the assessment and management of these patients. However, the optimal timing for such imaging remains a matter of ongoing debate [6,7].

While early brain computed tomography (CT) scans can identify acute intracranial pathologies or early signs of cerebral edema [6,8,9], their predictive value in assessing long-term neurological prognosis may be limited [7,10]. A contributing factor to this limitation is that signs of HIBI with subsequent cerebral edema after CA may not become evident until several hours to days post-event [5]. By then, initial CT scans might appear deceptively normal and not show an apparent indication for outcome.

Signs indicative of brain edema encompass the effacement of extracerebral fluid spaces such as the sulci or the perimesencephalic cisterns. Additionally, the reduction in grey-white matter differentiation between cortical or basalganglia regions and the surrounding white matter serves as a significant marker. Quantification of density, often expressed as the Grey-White Matter Ratio (GWR), has been a focus in numerous studies. It serves as a basis for neurological prognostication post-CA exhibiting a range of outcomes—some promising, while others less so [11,12,13]. Analogous to observations following stroke, alterations in grey and white matter densities over time after CA can be expected in line with the pathophysiological context of HIBI [5,7]. Therefore, recognition of signs of HIBI or markers indicative of its development in early post-CA CT may often be challenging for radiologists. Methods relying on conventional measurements of gray-white matter ratio densities offer no added value in situations where these densities are fundamentally still normal. Given this, there is a pressing need for more sophisticated tools that can detect subtle early changes indicative of HIBI and the risk of brain edema occurrence.

Radiomics is a field in contemporary radiology that involves the extraction of a vast number of quantitative features from radiological images, converting these images into data. Then, commonly, machine learning algorithms can be trained to give predictions based on these image features about clinically relevant information. Through this, radiomics-based algorithms have been utilized in various clinical scenarios, allowing for deeper insights into disease characteristics often imperceptible to the human eye [14,15,16]. In the context of post-CA, radiomics can potentially identify subtle changes in early brain CT scans, serving as harbingers for the eventual development of cerebral edema and HIBI in the post-CA cerebral assessment. To date, this approach has not yet been explored in this specific context. By utilizing quantitative imaging features, radiomics provides a significant advantage in identifying patterns that go beyond traditional density ratios while being less complex and more interpretable compared to some advanced AI-driven solutions. Radiomics, therefore, could provide a suitable framework for enhanced prognostic models.

By exploring a radiomics feature-based machine learning algorithm to predict the onset of cerebral edema from early normal appearing CT scans after CA, this study is the first to apply radiomics methodology to this specific clinical problem. It investigates whether subtle features in these images, imperceptible to the human eye, can be identified and utilized and beyond, whether focusing on possibly vulnerable regions, specifically the basalganglia region, is advantageous. This research seeks to close the gap between the utility of early CT scans, used to assess acute pathological changes, and delayed CT imaging, employed for neuroprognostication. Findings in this area could pave the way for improved early identification of at-risk patients, which could facilitate proactive interventions, such as intensified monitoring or neuroprotective strategies and ultimately improve patient outcomes.

## 2. Materials and Methods

### 2.1. Study Design

**Figure 1:**
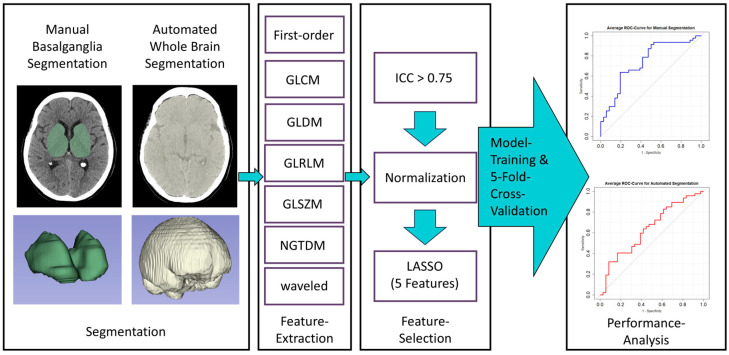
Illustration of the study design.

This retrospective study assesses patients who experienced CA and underwent resuscitation with subsequent return of spontaneous circulation (ROSC). The study design and protocols were approved by the institutional ethics committee. The need for patient consent was waived. The overarching goal was to predict the onset of brain edema by using a machine learning model trained on radiomics feature data from immediate post-ROSC brain CT scans. The study design is illustrated in Figure 1.

### 2.2. Cohort Identification

For this study, patients between June 2016 and June 2023 were considered. They were included if they suffered from a CA followed by ROSC, underwent CT imaging within 6 h post-ROSC, showed no acute findings or signs of brain edema in the initial brain CT, and had a follow-up brain CT between 1 to 14 days post-ROSC.

The initial brain CTs as well as the follow-up brain CTs were evaluated by a board-certified radiologist with access to the study reports.

**Figure 2:**
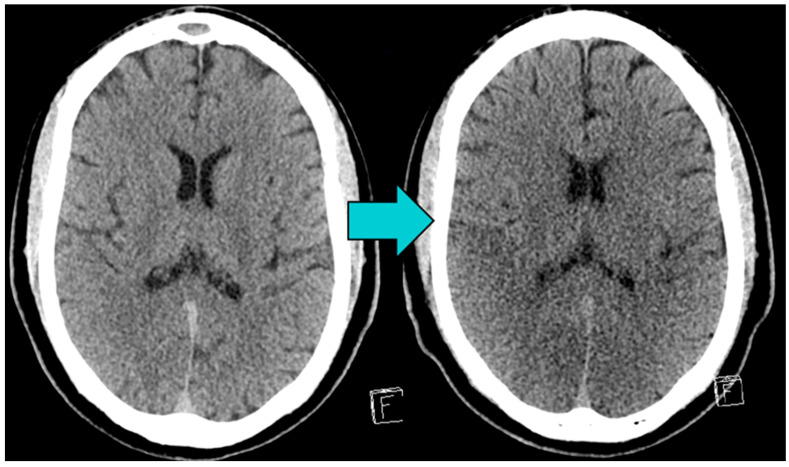
Images from a patient who suffered cardiac arrest (CA) and return of spontaneous circulation (ROSC). The initial unenhanced brain CT, taken < 6 h after ROSC (**left image**), shows no acute pathologies and no early signs of hypoxic-ischemic brain injury (HIBI). The follow-up brain CT (**right image**), taken 3 days after ROSC, shows global brain edema with diminished grey-matter white-matter differentiation and narrowed liquor rooms as signs of HIBI.

Patients were excluded if they presented with significant chronic brain defects (e.g., post-ischemic), large calcifications of the basalganglia, or if their CT scans were of poor image quality, e.g., due to artifacts. Exemplary initial and follow-up brain CTs of an included patient are shown in Figure 2.

### 2.3. CT-Protocol

As per institutional protocol, patients experiencing CA and ROSC receive an immediate whole-body CT, which includes an unenhanced brain scan. All Scans were conducted using a 64-detector row CT scanner (Definition AS^®^, Siemens Healthineers, Germany). Acquisition parameters for brain scans were: 0.6 mm collimation, a 1 s gantry rotation time, and a 0.55 pitch. 370 mAseff was used at a tube voltage of 120 kV. Scans were taken in the caudocranial direction. Images were reconstructed with a 3 mm slice thickness and a H37s Kernel.

### 2.4. Image Segmentation and Feature Extraction

Immediate post-ROSC CT images were segmented using 3DSlicer (v5.2.2, www.slicer.org (last accessed on 6 January 2025)) software. Two segmentation approaches were employed: manual segmentation of the basalganglia and automated whole-brain segmentation. For manual basalganglia segmentation, segmentation boundaries were defined using an anatomically based approach. Regions of interest were manually delineated on each axial slice to encompass the caudate nucleus, the thalamus, as well as the globus pallidus and putamen. These structures also served as the region of interest boundaries The internal capsule and the intervening white matter located between these structures were included in the region of interest. Automated whole-brain segmentation was performed using the TotalSegmentator plugin [17]. To refine the automated segmentation, threshold-based skull stripping was applied, ensuring that bone was not mistakenly segmented. Exemplary segmentations are illustrated in Figure 1.

Feature extraction was executed using the Pyradiomics (v.3.0.1) package within Slicer. Voxel size was adjusted to 1 × 1 × 1 mm and bin width was set to 25. This process led to the extraction of 837 features, encompassing first-order features, gray-level cooccurrence matrix (GLCM), gray-level dependence matrix (GLDM), gray-level run length matrix (GLRLM), gray-level size zone matrix (GLSZM), neighboring gray-tone difference matrix (NGTDM), and wavelet-based features. To assess the consistency of feature extraction, 18 CTs were randomly chosen and segmented again after a time interval of at least two months.

### 2.5. Feature Selection and Model Training

Feature reliability was first evaluated using the inter-class correlation coefficient (ICC). Any features with an ICC value below 0.75 were removed. The retained features underwent normalization using a min-max approach. Subsequently, LASSO regression was utilized, adjusting the Lambda value to retain only 5 features for each segmentation technique, reducing overfitting risks.

After initial explorations of the feature data, a logistic regression model was trained based on the identified features for each segmentation method. The training incorporated a 5-fold cross-validation, and results were summarized with average ROC curves and AUC values as well as average specificity and sensitivity.

All analyses were performed using R version 4.2.2 (R Foundation for Statistical Computing, Vienna, Austria) and Excel (Microsoft, Redmond, WA, USA).

## 3. Results

**Figure 3:**
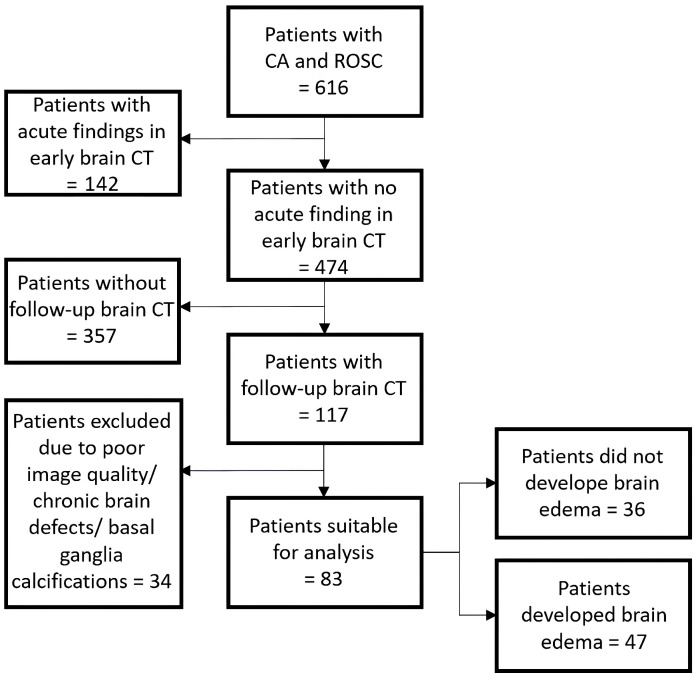
Flowchart to illustrate cohort identification in patients who suffered from cardiac arrest (CA), achieved a return of spontaneous circulation (ROSC), and received an early brain CT.

A total of 616 patient CTs with ROSC were reviewed. Of those, 474 appeared normal on the initial scan. A total of 117 of those had a follow-up CT within the following 1–14 days. Of the remaining 117, 34 had to be excluded due to significant chronic defects of brain parenchyma, large calcifications of the basal ganglia, or poor image quality due to artifacts (Figure 3).

Analysis was performed on the remaining 83 patients. Patients were 65 (±13.3) years old on average. A total of 19 of 83 were women. Follow-up CT was performed after an average of 5.2 (±2.9) days. 47/83 Patients had signs of brain edema in the follow-up CT scan.

### 3.1. Feature Selection

**Figure 4:**
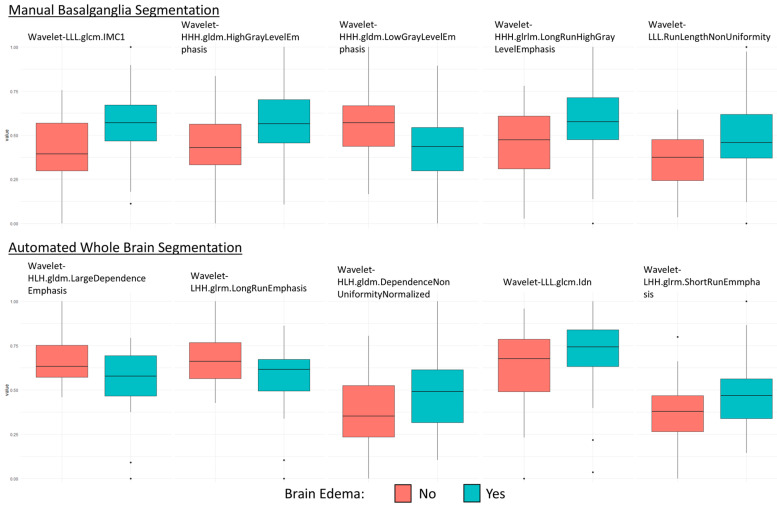
Boxplots of the used features from each segmentation method.

A total of 837 features were extracted for each segmentation technique. A total of 561 features were reproducible in the BG group and 703 features in the TotalSegmentator group, respectively. LASSO Regression identified five features in each segmentation group (Figure 4).

Basalganglia:

wavelet-HHH.gldm.HighGrayLevelEmphasis, wavelet-HHH.gldm.LowGrayLevelEmphasis, wavelet-HHH.glrlm.LongRunHighGrayLevelEmphasis, wavelet-LLL.glcm.Imc1 & wavelet-LLL.glrlm.RunLengthNonUniformity.

Totalsegmentator:

wavelet-LHH.glrlm.LongRunEmphasis, wavelet-LHH.glrlm.ShortRunEmphasis, wavelet-HLH.gldm.DependenceNonUniformityNormalized, wavelet-HLH.gldm.LargeDependenceEmphasis & wavelet-LLL.glcm.Idn.

### 3.2. Model Performance

The model with manual BG segmentation had an average AUC value of 0.76, an average sensitivity of 0.59, and an average specificity of 0.78.

**Figure 5:**
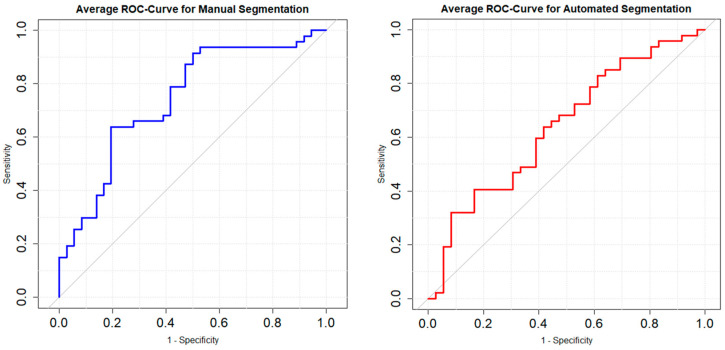
Average ROC curves of the model with manual basalganglia segmentation (**left**) and automated whole-brain segmentation (**right**).

The model with the automated whole-brain segmentation had an average AUC value of 0.66, an average sensitivity of 0.49, and an average specificity of 0.68. Average ROC curves are illustrated in Figure 5.

## 4. Discussion

Early post-CA CT scans are commonly used to detect acute brain pathologies such as stroke, bleeding, or early signs of brain edema. They also hold promising potential for predicting clinical progression and neurological outcomes. The grey-white matter ratio is typically a significant factor in interpreting CT scans in this context. However, reliable prognostication often requires approaches conducted within a week after CA [18]. Studies suggest that waiting longer after ROSC improves the accuracy of predictions in brain CT scans [7,19]. MRI also holds promise in the prediction of hypoxic-ischemic brain damage and neuroprognostication [20,21,22]. However, initial CT likely plays a more prominent role in the early emergency setting here due to its ability to be performed rapidly, its widespread accessibility, and its utility in identifying additional pathologies following cardiac arrest [9]. Therefore, enhancing the informative value of early brain CTs after ROSC proves to be advantageous.

This study aimed to predict the occurrence of brain edema after CA based on early post-CA brain CTs using a machine-learning approach. Among the 474 brain CTs without acute findings initially considered, 83 met the criteria for analysis, reflecting the challenges in patient selection due to missing follow-up CT and various brain-related factors or image quality issues. The occurrence of brain edema in 47 out of 83 patients during follow-up scans highlights the prevalence of this post-arrest complication, indicating its substantial occurrence in this cohort. Still, it is crucial to interpret this number with caution, considering the selection bias in this methodology.

The presented approach involved feature extraction with both manual basalganglia and automated whole-brain segmentation techniques and training of logistic regression models. Given the concept of selective vulnerability of different brain structures to hypoxia [23], the basalganglia appear to have a high ischemic vulnerability to hypoperfusion [24]. Previous studies suggest that the basal ganglia can serve as a sentinel site for detecting ischemic pathologies after resuscitated cardiac arrest [25]. Therefore, this analysis focused on this particular area. Logistic regression was chosen due to its simplicity and robustness as well as its suitability for relatively small datasets.

Model performance analysis indicated varying degrees of predictive capability between the two segmentation approaches. The model utilizing manual basalganglia segmentation exhibited superior performance, with an average AUC of 0.76, a sensitivity of 0.59, and a specificity of 0.78. On the other hand, the model relying on automated whole-brain segmentation showed comparatively lower performance metrics, with an average AUC of 0.66, sensitivity of 0.49, and specificity of 0.68.

Especially the performance of the model utilizing basalganglia segmentation indicates that the risk of development of brain edema may be assessable based on early CT scans, even if they appear normal to the radiologist’s eye. A machine learning-based model could aid in cases where the initial CT scans appear normal to radiologists, identifying patients at risk for brain edema and possibly aiding clinicians with therapy guidance. Still, model performance needs to be further refined.

The differences in model performance suggest the potential advantages of basalganglia segmentation in capturing nuanced changes relevant to post-CA brain pathology compared to the broader automated whole-brain segmentation. This could be attributed, in part, to the co-segmentation of less relevant brain regions, which may dilute the predictive power of crucial features. Moreover, the basal ganglia region appears particularly significant in this context. The results contribute additional evidence to the existing literature, which suggests that future studies should focus on changes in this particular area to improve neuroprognostication after CA. Furthermore, the results underscore the need for more refined automated segmentation tools capable of accurately isolating specific brain regions, as manual segmentation is both time consuming and potentially inconsistent. This is further highlighted by the disparity in the number of reproducible features between the two methods (561 vs. 703).

Mansour et al. [26] showcased impressive results in a similar study design. Their recently published study describes an approach utilizing deep transfer learning on early head-CT scans, initially considered normal, to forecast HIBI indicated as brain edema in follow-up scans. Similar to this study, their model was also trained on normal-appearing early brain CTs. Their model exhibited high AUC value, specificity, and sensitivity and showed that deep learning accurately predicted the emergence of brain edema. These findings indicate that even while appearing normal, there may be quantifiable changes in appearance in the early brain CT after ROSC, which can be used to give a risk stratification. However, these remarkable results need to be interpreted with caution. A pertinent commentary on these studies highlights concerns [27]. The utilized deep learning-based algorithm may be challenging to explain and interpret. Additionally, these algorithms tend to overfit, especially with smaller datasets [27,28]. The extraordinary accuracy demonstrated by this study may not necessarily be generalizable. Nonetheless, their findings remain relevant.

Kawai et al. [29] investigated predicting poor neurological outcomes post-CA using head CT scans taken shortly after resuscitation. They constructed an explainable artificial intelligence-based prognostic model, comparing its accuracy with the traditional GWR method. Analyzing 321 patients, their model, employing transfer learning on CT images, predicted outcomes at 1-month post-resuscitation. The accuracy achieved in this study may align with that of the presented study. Additionally, they showed the superiority of the results of the deep learning model over the GWR method. Their emphasis on explainability through heatmap visualization is a noteworthy advancement in making the model more interpretable and clinically relevant. Nevertheless, a crucial factor to consider when comparing the performance of the model to ours is that the study’s cohort might include patients with acute pathologies, perhaps even early brain edema with obvious changes in CT appearance. This could make the prediction of poor neurological outcomes easier for the model.

It is important to note that this study did not incorporate clinical parameters, as outcome prediction should include multiple diagnostic modalities [1]. Early predictors could include factors like initial rhythm, duration of resuscitation, age, gender, or laboratory parameters. These factors could also be incorporated into a machine learning algorithm to further refine the performance of the model [30]. Unfortunately, due to inconsistent documentation in the cohort, such an analysis could not be conducted. Nonetheless, when establishing similar studies, considering these parameters is crucial and could offer substantial benefits. The utilized approach is theoretically well-suited in this regard, as it is easily adaptable and amendable to incorporating clinical parameters.

Further, it is crucial to consider selection bias in this context. Many patients could not be included due to the absence of follow-up CT scans. Those patients who underwent follow-up CT scans likely exhibited some form of neurological symptoms or remained in a comatose state. However, establishing a definitive statement of whether or not the patient developed brain edema was essential. Therefore, having a follow-up examination was crucial. To address this issue, a prospective data collection method is preferable. This approach could involve the inclusion of clinical parameters and the prognostication of neurological outcomes.

Other key limitations of this study include its retrospective design, the relatively small patient sample, and data confined to a single clinic and single CT scanner. Optimally, the model should be validated with an external cohort.

## 5. Conclusions

Radiomics features from normal-appearing early brain CTs after CA and ROSC, together with a simple logistic regression model, might be a viable, explainable, and simple method to add additional information for predicting the development of brain edema. The area of basalganglia is of particular relevance in this context. Further refinement is still needed, for example, through the incorporation of available clinical information.

## Data Availability

The data presented in this study are available on reasonable request from the corresponding author. The data are not publicly available due to privacy or ethical restrictions.

## References

[1] Nolan J.P., Neumar R.W., Adrie C., Aibiki M., Berg R.A., Böttiger B.W., Callaway C., Clark R.S., Geocadin R.G., Jauch E.C. (2008). Post-cardiac arrest syndrome: Epidemiology, pathophysiology, treatment, and prognostication. A Scientific Statement from the International Liaison Committee on Resuscitation; the American Heart Association Emergency Cardiovascular Care Committee; the Council on Cardiovascular Surgery and Anesthesia; the Council on Cardiopulmonary, Perioperative, and Critical Care; the Council on Clinical Cardiology; the Council on Stroke. Resuscitation.

[2] Coute R.A., Nathanson B.H., Panchal A.R., Kurz M.C., Haas N.L., McNally B., Neumar R.W., Mader T.J., CARES Surveillance Group (2019). Disability-Adjusted Life Years Following Adult Out-of-Hospital Cardiac Arrest in the United States. Circ. Cardiovasc. Qual. Outcomes.

[3] Dragancea I., Rundgren M., Englund E., Friberg H., Cronberg T. (2013). The influence of induced hypothermia and delayed prognostication on the mode of death after cardiac arrest. Resuscitation.

[4] Sekhon M.S., Ainslie P.N., Griesdale D.E. (2017). Clinical pathophysiology of hypoxic ischemic brain injury after cardiac arrest: A “two-hit” model. Crit. Care.

[5] Elmer J., Callaway C. (2017). The Brain after Cardiac Arrest. Semin. Neurol..

[6] Çankaya Gökdere D., Emektar E., Çorbacıoğlu Ş.K., Yüzbaşıoğlu Y., Öztürk C., Çevik Y. (2022). The Role of Brain CT in Patients with Out-of-Hospital Cardiac Arrest with Return of Spontaneous Circulation. Am. J. Emerg. Med..

[7] Streitberger K.J., Endisch C., Ploner C.J., Stevens R., Scheel M., Kenda M., Storm C., Leithner C. (2019). Timing of brain computed tomography and accuracy of outcome prediction after cardiac arrest. Resuscitation.

[8] Reynolds A.S., Matthews E., Magid-Bernstein J., Rodriguez A., Park S., Claassen J., Agarwal S. (2017). Use of early head CT following out-of-hospital cardiopulmonary arrest. Resuscitation.

[9] Viniol S., Thomas R.P., König A.M., Betz S., Mahnken A.H. (2020). Early whole-body CT for treatment guidance in patients with return of spontaneous circulation after cardiac arrest. Emerg. Radiol..

[10] Hong J.Y., Lee D.H., Oh J.H., Lee S.H., Choi Y.H., Kim S.H., Min J.H., Kim S.J., Park Y.S. (2019). Grey-white matter ratio measured using early unenhanced brain computed tomography shows no correlation with neurological outcomes in patients undergoing targeted temperature management after cardiac arrest. Resuscitation.

[11] Scheel M., Storm C., Gentsch A., Nee J., Luckenbach F., Ploner C.J., Leithner C. (2013). The prognostic value of gray-white-matter ratio in cardiac arrest patients treated with hypothermia. Scand. J. Trauma Resusc. Emerg. Med..

[12] Lee B.K., Jeung K.W., Song K.H., Jung Y.H., Choi W.J., Kim S.H., Youn C.S., Cho I.S., Lee D.H. (2015). Prognostic values of gray matter to white matter ratios on early brain computed tomography in adult comatose patients after out-of-hospital cardiac arrest of cardiac etiology. Resuscitation.

[13] Na M.K., Kim W., Lim T.H., Jang B., Cho Y., Choi K.-S., Shin H.-G., Ahn C., Lee J., Kim J.G. (2018). Gray matter to white matter ratio for predicting neurological outcomes in patients treated with target temperature management after cardiac arrest: A systematic review and meta-analysis. Resuscitation.

[14] Rogers W., Seetha S.T., Refaee T.A.G., Lieverse R.I.Y., Granzier R.W.Y., Ibrahim A., Keek S.A., Sanduleanu S., Primakov S.P., Beuque M.P.L. (2020). Radiomics: From qualitative to quantitative imaging. BJR.

[15] Parekh V.S., Jacobs M.A. (2019). Deep learning and radiomics in precision medicine. Expert. Rev. Precis. Med. Drug Dev..

[16] Koçak B., Durmaz E.Ş., Ateş E., Kılıçkesmez Ö. (2019). Radiomics with artificial intelligence: A practical guide for beginners. Diagn. Interv. Radiol..

[17] Wasserthal J., Breit H.-C., Meyer M.T., Pradella M., Hinck D., Sauter A.W., Heye T., Boll D.T., Cyriac J., Yang S. (2023). TotalSegmentator: Robust Segmentation of 104 Anatomic Structures in CT Images. Radiol. Artif. Intell..

[18] Sandroni C., D’arrigo S., Cacciola S., Hoedemaekers C.W.E., Kamps M.J.A., Oddo M., Taccone F.S., Di Rocco A., Meijer F.J.A., Westhall E. (2020). Prediction of poor neurological outcome in comatose survivors of cardiac arrest: A systematic review. Intensive Care Med..

[19] In Y.N., Lee I.H., Park J.S., Kim D.M., You Y., Min J.H., Jeong W., Ahn H.J., Kang C., Lee B.K. (2022). Delayed head CT in out-of-hospital cardiac arrest survivors: Does this improve predictive performance of neurological outcome?. Resuscitation.

[20] Beekman R., Hirsch K.G. (2023). Brain imaging after cardiac arrest. Curr. Opin. Crit. Care.

[21] Lopez Soto C., Dragoi L., Heyn C.C., Kramer A., Pinto R., Adhikari N.K.J., Scales D.C. (2020). Imaging for Neuroprognostication After Cardiac Arrest: Systematic Review and Meta-analysis. Neurocrit. Care.

[22] Wijman C.A.C., Mlynash M., Caulfield A.F., Hsia A.W., Eyngorn I., Bammer R., Fischbein N., Albers G.W., Moseley M. (2009). Prognostic value of brain diffusion-weighted imaging after cardiac arrest. Ann. Neurol..

[23] Cervós-Navarro J., Diemer N.H. (1991). Selective vulnerability in brain hypoxia. Crit. Rev. Neurobiol..

[24] Payabvash S., Souza L.C., Wang Y., Schaefer P.W., Furie K.L., Halpern E.F., Gonzalez R.G., Lev M.H. (2011). Regional Ischemic Vulnerability of the Brain to Hypoperfusion. Stroke.

[25] Haglund M., Lindberg E., Englund E. (2019). Hippocampus and basal ganglia as potential sentinel sites for ischemic pathology after resuscitated cardiac arrest. Resuscitation.

[26] Mansour A., Fuhrman J.D., El Ammar F., Loggini A., Davis J., Lazaridis C., Kramer C., Goldenberg F.D., Giger M.L. (2022). Machine Learning for Early Detection of Hypoxic-Ischemic Brain Injury After Cardiac Arrest. Neurocrit. Care.

[27] Molinski N.S., Meddeb A., Kenda M., Scheel M. (2022). Comment on “Machine Learning for Early Detection of Hypoxic-ischemic Brain Injury After Cardiac Arrest”. Neurocrit. Care.

[28] Hawkins D.M. (2004). The problem of overfitting. J. Chem. Inf. Comput. Sci..

[29] Kawai Y., Kogeichi Y., Yamamoto K., Miyazaki K., Asai H., Fukushima H. (2023). Explainable artificial intelligence-based prediction of poor neurological outcome from head computed tomography in the immediate post-resuscitation phase. Sci. Rep..

[30] Dünser M.W., Hirschl D., Weh B., Meier J., Tschoellitsch T. (2023). The value of a machine learning algorithm to predict adverse short-term outcome during resuscitation of patients with in-hospital cardiac arrest: A retrospective study. Eur. J. Emerg. Med..

